# Characteristics of folic acid metabolism-related genes unveil prognosis and treatment strategy in lung adenocarcinoma

**DOI:** 10.1186/s12890-025-03694-x

**Published:** 2025-05-22

**Authors:** Yanting Dong, Xiaoyan Wang, Chuanchuan Dong, Peiqi Li, Zhuola Liu, Xinrui Tian

**Affiliations:** 1 Department of Respiratory and Critical Care Medicine, The Second Hospital of Shanxi Medical University, Taiyuan, China; 2 Beijing Health Vocational College, Beijing, China; 3 Clinical Medicine, The Second Hospital of Shanxi Medical University, Taiyuan, China; 4 Department of Geratology, The Second Hospital of Shanxi Medical University, Taiyuan, China

**Keywords:** Folic acid, Lung adenocarcinoma, Prognosis, Overall survival, Risk score

## Abstract

**Background:**

Lung adenocarcinoma (LUAD) is the most common subtype of lung cancer. Folic acid metabolism-related genes (FAMGs) have received increased attention because of their distinct role in DNA synthesis and repair. Nevertheless, the function of FAMGs in LUAD remains ambiguous.

**Methods:**

LUAD transcriptome data from GEO and TCGA were analyzed. Patients were classified into two clusters based on gene expression levels, revealing distinct overall survival (OS) outcomes. Common differentially expressed genes (DEGs) were identified between LUAD and normal tissues, as well as between the two clusters. A prognostic risk model was established using Cox regression analysis to predict outcomes of LUAD patients and was validated with Kaplan-Meier and ROC curve analysis. Clinical correlations and enrichment analyses were carried out to explore the functions of DEGs and their associations with clinical characteristics of LUAD patients. The tumor microenvironment and drug sensitivity were evaluated between two risk subgroups. Moreover, expression levels of prognostic genes were validated across datasets using the Wilcoxon-test.

**Results:**

The study identified seventy-seven common DEGs and nine prognostic genes (*ANLN*,* PLK1*,* DLGAP5*,* PRC1*,* CYP4B1*,* MKI67*,* KIF23*,* BIRC5*,* TK1*). The risk model could effectively predict the prognosis of LUAD patients. Clinical correlation analysis revealed that age, pathologic-T, pathologic-N, and tumor stage were significantly correlated with the risk score. Enrichment analysis showed that DEGs between the two risk subgroups were predominantly enriched in cell cycle and cellular senescence pathways. Differences in immune cell infiltration and immunotherapy markers were markedly noted between the two risk subgroups. Drug sensitivity analysis disclosed significantly diverse responses to sixty-eight drugs between the two risk subgroups. Consistent expression tendencies of prognostic genes were observed across datasets.

**Conclusion:**

The prognostic model based on FAMGs demonstrates considerable potential for guiding diagnosis and clinical management of LUAD patients.

**Supplementary Information:**

The online version contains supplementary material available at 10.1186/s12890-025-03694-x.

## Introduction

Lung adenocarcinoma (LUAD) is the most frequently occurring subtype of lung cancer, accounting for over 60% of non-small cell lung cancers [1]. Attributed to its exceptionally high lethality and comorbidity, LUAD constitutes a substantial global contributor to cancer-related mortality [2]. Despite the remarkable progress in the diagnosis, monitoring and immunotherapy of LUAD, its overall survival (OS) rate remains unsatisfactory [3]. The development and pathogenesis of LUAD are complex, involving genetic and environmental factors [4]. In recent years, numerous genetic alterations have been identified to be associated with the development of LUAD, and several risk models have been constructed to explore its prognosis and therapeutic significance [5–7]. Nevertheless, the mechanisms of LUAD remain elusive, and the personalized treatment strategy still confronts substantial challenges. In addition to genetic factors, metabolism and related transduction pathways have been demonstrated to be associated with tumor occurrence, and metabolism reprogramming seems to be prevalent in tumor cells to sustain their biological processes and malignant proliferation [8]. He et al. identified 13 metabolic genes as independent factors and established a prognostic prediction model based on the metabolism-related gene signature for LUAD [9]. Metabolic pathways may influence tumor progression through interaction with the tumor microenvironment or by altering metabolic-driven gene regulations [10]. Consequently, a better comprehension of the cancer metabolism pathway and exploration of key metabolic biomarkers may provide significant guidance for improving the outcome of LUAD.

Among all the metabolic processes, folic acid and the related one-carbon metabolism are essential for DNA synthesis, repair, and methylation in human cells [11]. It’s widely acknowledged that reduced folic acid intake during pregnancy is associated with an increased risk of neural tube defects [12, 13]. In adults, folic acid has been considered as a potential protective agent against various cancers. According to epidemiological researches, low levels of folic acid were associated with malignancies of the cervix, colorectum, lung, esophagus, brain, pancreas, and breast [14–16]. Furthermore, other studies have also revealed the correlation between serum folic acid levels and the risk of lung cancer. For instance, Durda et al. [17] measured the serum folic acid levels in 366 lung cancer patients and 366 control subjects. The results indicated that lower concentrations of serum folic acid were associated with a higher risk of lung cancer. Swartz et al. investigated the roles of folic acid status, nutrition, genes, and gene-nutrient interactions in the folic acid metabolic pathway on lung cancer risk, suggesting that the impact of dietary interventions on lung cancer risk may be modulated by genotypes in key folic acid metabolic genes [18]. The close association of folic acid with cancer risk might be attributed to its specific function of transferring one-carbon units in the synthesis of S-adenosylmethionine (SAdoMet), a methyl donor for the biological methylation of DNA, RNA, proteins, etc., and de novo synthesis of oxyribonucleotide triphosphate [11, 19–21]. Consequently, low folic acid intake may result in folic acid deficiency and disturbances of folic acid-related metabolisms, and cause DNA damage, alterations in chromosome integrity, and disruptions of DNA repair. Previous studies have discovered that inaccurate DNA methylation leads to the instability of DNA and may occur at the onset of lung cancer [22, 23]. These might be the underlying mechanisms for the lung cancer risk associated with folic acid deficiency in humans.

Therefore, further exploration of more effective prognostic biomarkers and discovery of the potential molecular mechanisms related to folic acid metabolism are of great significance in the clinical diagnosis and therapy of LUAD. The current study is one of the first ones to focus on the role of folic acid metabolism-related genes (FAMGs) in LUAD by integrating and analyzing transcriptome data from GEO and TCGA datasets. We identified multiple prognostic genes and constructed an effective prognostic risk model based on Cox regression analysis. The model was further validated and verified to be stable and possess ideal prognostic performance. The present study not only offers new insights into the molecular mechanisms of LUAD, but also demonstrates a significant potential of FAMGs-related prognostic model in guiding the diagnosis and clinical management of LUAD patients.

## Materials and methods

### Data collecting

The Cancer Genome Atlas (TCGA) and the Gene Expression Omnibus (GEO) databases provided transcriptomic data and clinical information on LUAD. The TCGA-LUAD dataset, consisting of 513 tumor and 59 normal samples, was utilized as the training set for consensus clustering analysis and differential expression analysis. Meanwhile, a total of 334 LUAD samples with clinical information were obtained for clinical relevance analysis in the TCGA-LUAD dataset. The GSE31210 dataset, which contains 226 tumor and 20 normal samples, was utilized as the validation set. Moreover, 15 folic acid metabolism-related genes (FAMGs) were obtained from the Kyoto Encyclopedia of Genes and Genomes (KEGG) database (https://www.kegg.jp/), the adhesion molecule with Ig-like domain 2 (AMIGO2) database (https://www.lsbio.com/targets/amigo2/g 129569), and related literature. The selection process was described as follows. In the KEGG database, the keyword “folic acid metabolism” or related words were searched, and the folic acid-related metabolic pathway diagram was obtained. Furthermore, the enzymes and genes directly related to folic acid metabolism were identified. In the AmiGO 2 database, the GO terms related to folic acid metabolism, such as “folic acid biosynthesis” and “folic acid metabolic process”, were searched, and the genes with these GO annotations were selected. In PubMed, keywords related to folic acid metabolism, such as “folic acid metabolism” and “folic acid gene”, were searched, and the highly related literature were selected. And genes mentioned in the literature were identified as being directly related to folic acid metabolism. These genes selected from KEGG, AmiGO 2, and PubMed were merged while removing redundant genes. Finally, 15 essential genes related to the folic acid metabolic pathway were selected [24].

### Differential expression analysis

Firstly, differentially expressed genes (DEGs) between tumor and normal samples were identified in the TCGA-LUAD dataset using the “limma”-package (v 3.42.2) [25] with adj.*P* < 0.05 and|logFC|>1. Meanwhile, 513 LUAD patients were consistently clustered using the “ConsensusClusterPlus”-package (v 1.54.0) [26]. Subsequently, the same operation was performed in the different clusters to screen for DEGs, and the correlation coefficients and p-values between FAMGs and screened DEGs were calculated. Expression matrices of FARGs and 162 differentially expressed genes were successively extracted from TCGA-LUAD transcriptome data, and the Spearman method was used to obtain the correlation coefficient and P-value. These were then screened based on the threshold criteria of P-value < 0.05 and|cor| > 0.6 to obtain DEGs that were significantly correlated with FAMGs. Subsequently, the DE-FAMGs obtained above were intersected with the DEGs obtained in the TCGA-LUAD dataset through the “VennDiagram”-package (v 1.6.20) [27] to obtain the common DEGs. Next, the “clusterProfiler”-package (v 4.14.1) [28, 29] was utilized to conduct Gene Ontology (GO) functional and KEGG pathway enrichment analysis for the common DEGs.

### Construction and validation of a prognostic risk model for LUAD patients

Taking TCGA-LUAD as the training set, univariate Cox proportional hazards regression analysis was conducted on the common genes. Based on the expression levels of the screened genes, the patients were divided into two groups: high expression and low expression groups. Subsequently, Kaplan-Meier (KM) survival analysis was carried out. The relevant variables obtained from the survival analysis were incorporated into the multivariate Cox analysis. And subsequently, the optimal regression equation was derived using the function step() in stepwise regression. A prognostic risk model was constructed via the “survival”-package (v 3.2-3) [30]. Afterwards, LUAD patients in the training and validation sets were classified into high- and low-risk subgroups based on the expression of these survival-related DEGs. Additionally, receiver operating characteristic (ROC) and Kaplan-Meier (K-M) curves were delineated using the “survivalROC”-package (v 6.5-0) [31] to assess the predictive accuracy and sensitivity of the risk model.

### Clinical correlation and independent prognostic analyses

To further illuminate the relationship between the risk model and clinicopathological features, we investigated the association between diverse characteristics (age, sex, stage, pathologic-M/N/T) and the risk model in 334 samples from TCGA-LUAD dataset. Subsequently, these features and the risk score were integrated into Cox analysis to identify independent prognostic predictors. Furthermore, a nomogram was constructed via the “rms”-package [32] to predict the survival of LUAD patients. In addition, the calibration and decision curves were plotted to evaluate the reliability of the predictions of this nomogram model.

### Enrichment analysis between the two risk subgroups

To explore the underlying biological processes of the risk model, genes associated with the risk score were identified using Spearman (|R|>0.5). Subsequently, “clusterProfiler”-package (v 4.14.1) was utilized to conduct GO functional and KEGG pathway enrichment analysis on these genes associated with the risk score.

### Tumor microenvironment analysis

To investigate the immune cell infiltration between the two risk subgroups from the TCGA-LUAD dataset, the CIBERSORT method [33] was utilized. Subsequently, the risk score and seven metagenic clusters were evaluated for correlation using the “correlogram” R package (v 1.14) [34].

### Immunotherapy and drug sensitivity analysis

To explore the association between the risk model and the immunotherapy response, we investigated the differences in tumour mutation burden (TMB), neoantigen number, clonal neoantigen number, and subclonal neoantigen number between the two risk subgroups in TCGA-LUAD. Moreover, tumor immune dysfunction and exclusion (TIDE) was used to evaluate the tumor immune evasion ability and predict the response to immune checkpoint inhibitor between the two risk groups. Furthermore, through the Genomics of Drug Sensitivity in Cancer (GDSC) database (https://www.cancerrxgene.org/), the pRRophetic algorithm [35] was adopted to predict the sensitivity of LUAD patients to drugs in the two risk subgroups.

### Statistical analysis

The statistical analyses and visualization of the results were conducted using R software (v 4.0.3). The Wilcoxon-test was utilized to validate the expression levels of the nine-risk model genes between the two risk subgroups. The Spearman method was adopted to calculate the correlation coefficient between FAMGs and DEGs. A *P*-value less than 0.05 was regarded as a statistically significant difference.

## Results

### Different folic acid metabolism molecular subtypes in LUAD patients

**Fig. 1:**
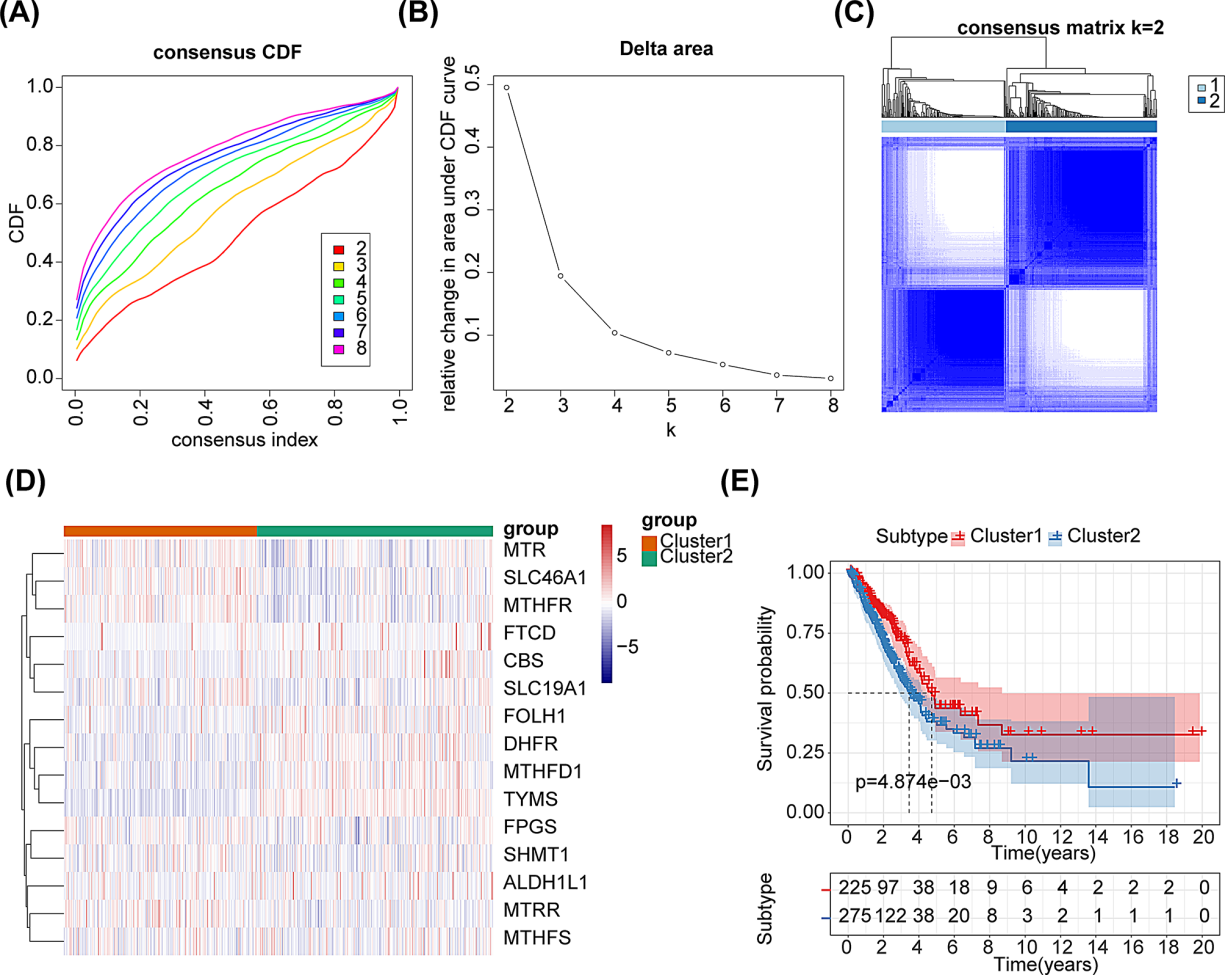
Consensus clustering analysis. (**A**) Cumulative distribution function (CDF) curve for k = 2–8. (**B**) Delta area curve of consensus clustering. (**C**) Consensus clustering matrix for k = 2. (**D**) Heatmap of gene expression levels of FAMGs in the two clusters. Orange represented cluster 1, and green represented cluster 2. Red indicated high expression, while blue indicated low expression. (**E**) K-M survival analysis of the two subtypes

Consensus clustering analysis was conducted to explore novel subtypes of folic acid-related molecules in LUAD patients. Ultimately, when k = 2, LUAD patients were classified into 2 clusters in accordance with FAMGs (Figs. 1A-C). A heatmap of the expressions of FAMGs between the two clusters were presented in Fig. 1D. K-M curve was then utilized to analyze the OS of the two subtypes, and the results indicated that there was a significant difference in OS between the different subtypes (*P* < 0.05), and that cluster 2 had a higher survival rate than cluster 1 (Fig. 1E).

### Selection and enrichment analysis of DEGs

**Fig. 2:**
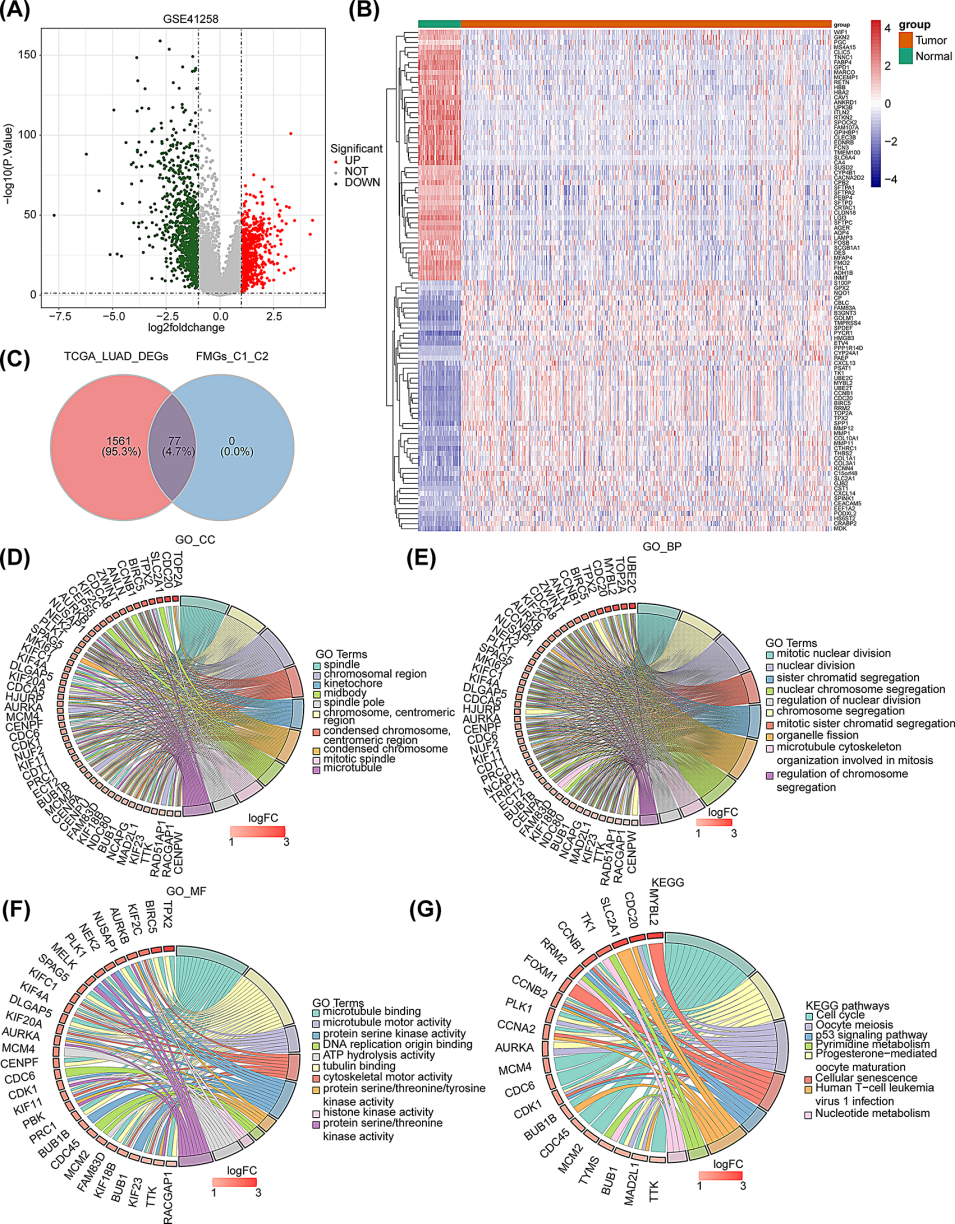
Screening and enrichment analysis of common DEGs. (**A**) Volcano plot depicted DEGs between LUAD and normal samples in TCGA-LUAD. Red: upregulation; green, downregulation; grey: not significant. (**B**) Heatmap depicted top 50 up-/ down-regulated DEGs between LUAD and normal samples in TCGA-LUAD. (**C**) Venn diagrams revealed 77 common DEGs. (**D**-**F**) Chord diagrams illustrate GO analysis for CC (**D**), BP (**E**), and MF (**F**) on common DEGs. (**G**) Chord diagram of KEGG analysis on common DEGs

To explore potential FAMGs in LUAD, we identified DEGs between LUAD and normal groups in TCGA-LUAD dataset. A total of 1,638 DEGs were obtained, with 944 genes being down-regulated and 694 genes being up-regulated (Fig. 2A). The expression patterns of the top 50 up-/down-regulated DEGs of the dataset were shown in Fig. 2B. Meanwhile, we performed the same operation in both FAMGs subtypes, and 162 DEGs (80 up-regulated and 82 down-regulated) were screened. Subsequently, the correlation coefficients between the FAMGs and these 162 DEGs (Cluster1 vs. Cluster2) were computed using the Spearman method, and 77 DE-FAMGs were obtained. Finally, 77 common DEGs were obtained by the intersection of DEGs in the TCGA-LUAD dataset and DE-FAMGs for subsequent analysis (Fig. 2C). Moreover, we implemented GO and KEGG analysis to determine the effects of the 77 common DEGs on biological processes. The results of GO enrichment analysis indicated that common DEGs were mainly involved in 10 respective terms such as condensed chromosome, and mitotic spindle in the cellular component (CC) (Fig. 2D), mitotic nuclear division, organelle fission, etc. in the biological process (BP) (Fig. 2E) and cytoskeletal motor activity, tubulin binding, and histone kinase activity in the molecular function (MF) (Fig. 2F). In addition, in the KEGG pathways, a total of 17 signaling pathways, including cell cycle, cellular senescence, and other pathways, were shown to be significantly associated with these common DEGs (Fig. 2G).

### Construction and verification of the folic acid-related risk model for LUAD patients

**Fig. 3:**
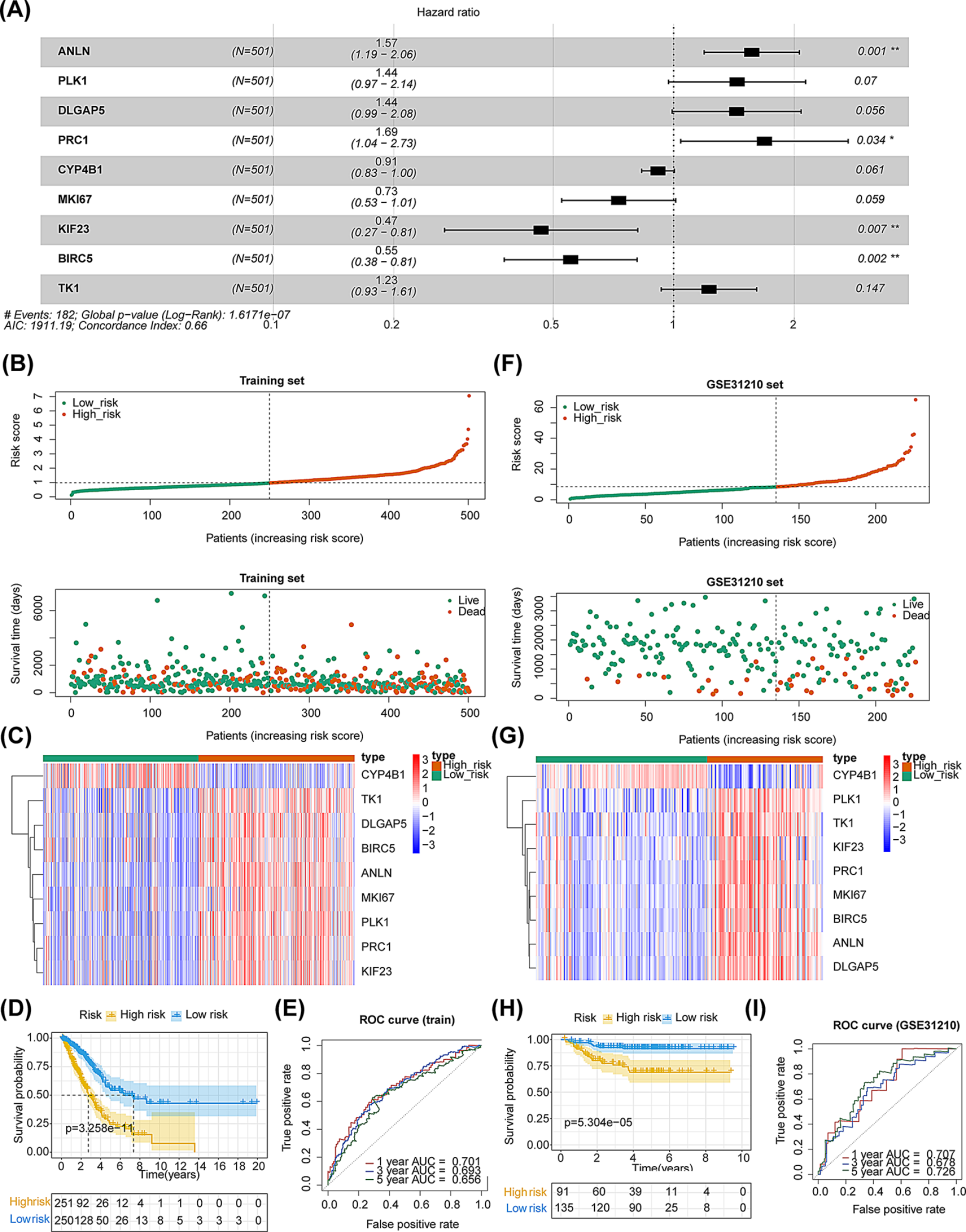
Creation and verification of the folic acid-related risk model. (**A**) Multivariate Cox regression analysis. (**B**) Risk score distribution and survival time distribution in TCGA-LUAD. (**C**) Nine genes distribution in TCGA-LUAD. (**D**) K-M survival analysis in TCGA-LUAD. (**E**) ROC curves for 1-, 3-, 5-year in LUAD samples in TCGA-LUAD. (**F**-**I**) Verification in the GSE31210 dataset: risk score and survival time distributions (**F**), genes distribution (**G**), K-M survival analysis (**H**), and ROC curves (**I**)

Based on univariate Cox analysis, we discovered that 46 out of 77 DEGs were significantly associated with OS (*P* < 0.001). LUAD patients were subsequently categorized into high- and low-expression groups for K-M survival analysis based on the expression of these 46 genes, and 25 of these DEGs were selected for the construction of a multivariate Cox regression analysis (Supplementary Fig. 1, Supplementary Table 1). A total of 9 DEGs (*ANLN*,* PLK1*,* DLGAP5*,* PRC1*,* CYP4B1*,* MKI67*,* KIF23*,* BIRC5*, and *TK1*) were obtained through multivariate Cox regression analysis (Fig. 3A). Subsequently, the risk score for each LUAD patient within the TCGA-LUAD dataset was computed, and thereafter, they were categorized into high- and low-risk subgroups according to the truncation value (0.97) (Figs. 3B-C). The OS curve outcomes demonstrated a significant difference in patient survival between the two risk subgroups (*P* < 0.05), and high-risk patients presented a lower survival rate (Fig. 3D). ROC analysis indicated that the AUC values of the risk model at 1-, 3-, and 5-year in the TCGA-LUAD dataset were all greater than 0.6, indicating an excellent predictive performance of the risk model (Fig. 3E). Besides, the risk model was validated in the GSE31210 dataset, and the obtained results were consistent with those in the TCGA-LUAD dataset (Figs. 3F-I).

### Risk score and clinical correlation analysis

**Fig. 4:**
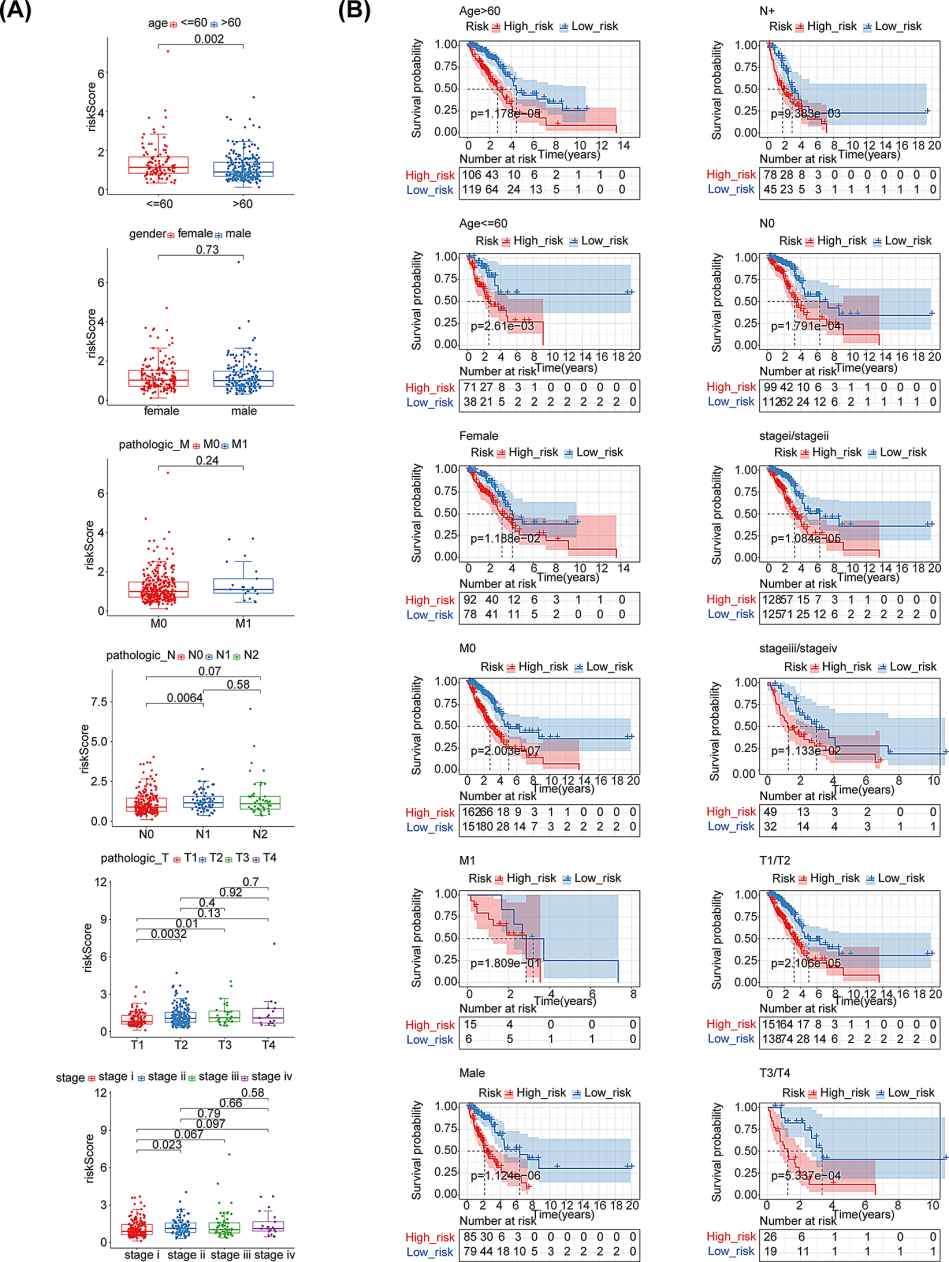
Correlation analysis between risk score and clinical characteristics. (**A**) The risk score in different clinical subgroups. (**B**) Stratified survival analysis for various clinical subgroups

To investigate the correlation between clinical pathological features and the risk score, 334 TCGA-LUAD samples with clinical information were used for correlation analysis. The results revealed that the risk score was significantly different from age, pathologic-T, pathologic-N, and tumor stage (Fig. 4A). Stratified analyses were performed to obtain a deeper understanding of the correlation between the two risk subgroups. Results indicated that Age > 60, Age ≤ 60, female, male, T1/T2, T3/T4, M0, N0, N+, stage I-stage IV all had significant differences between the two risk subgroups (*P* < 0.05) (Fig. 4B).

### Risk score was an independent risk factor for LUAD patients

**Fig. 5:**
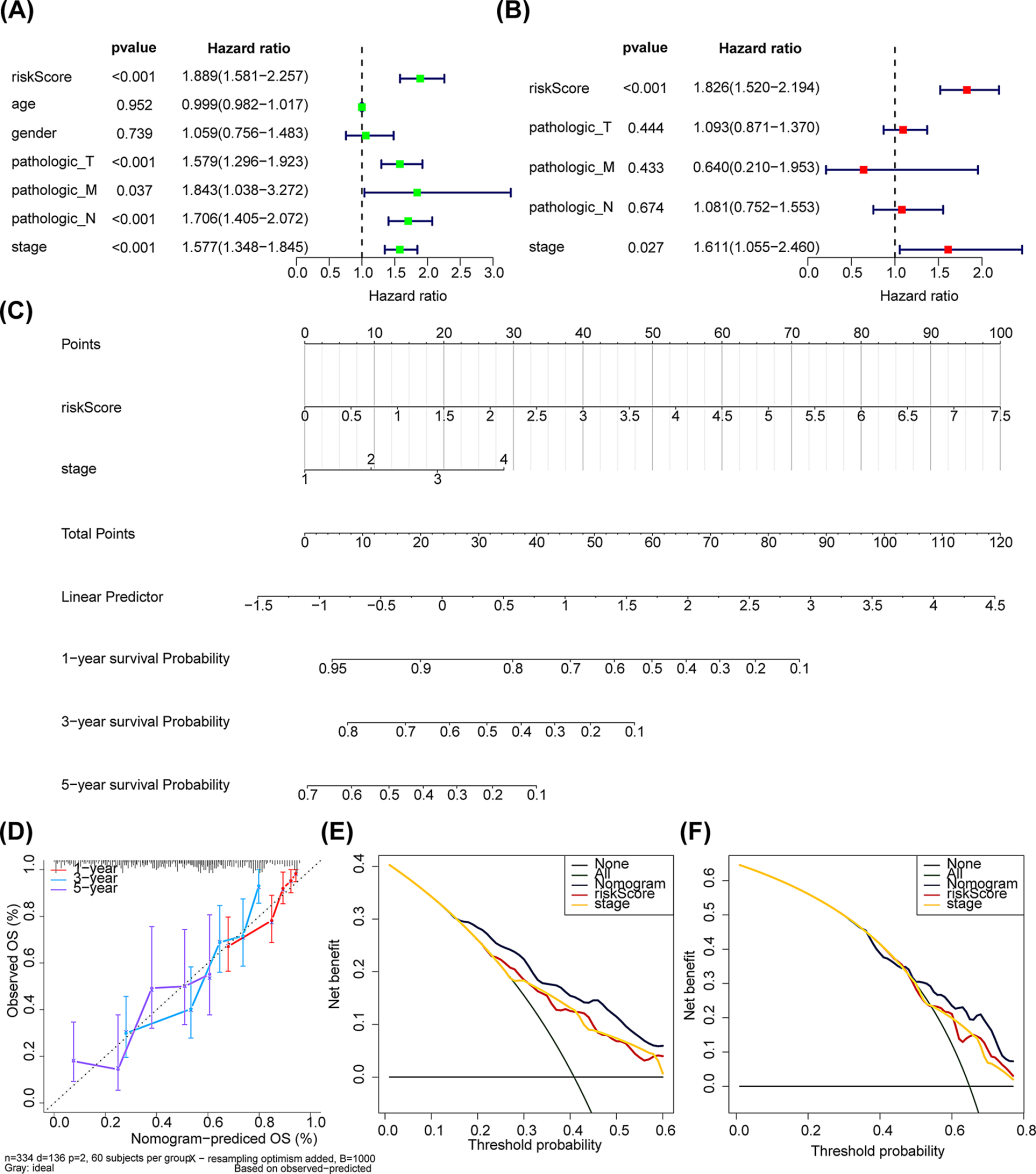
Independent prognostic analysis and nomogram creation. (**A**) Univariate Cox regression analysis of risk score and clinical factors. (**B**) Multivariate Cox regression analysis. (**C**) Nomogram for predicting 1-, 3‐, and 5-year survival probability of LUAD patients. (**D**) Calibration curve of the nomogram. (**E**-**F**) Decision curve of the nomogram for predicting 3‐, and 5-year survival probability

Four significant independent prognostic factors (stage, pathologic-T/N/M) were obtained through univariate Cox analysis (Fig. 5A). Subsequently, these 4 independent risk factors associated with a *P* value < 0.05 were further analyzed through multivariate Cox analysis. The results indicated that the risk score (*P* < 0.05) was a more reliable independent prognostic factor for LUAD (Fig. 5B). Furthermore, the nomogram model was constructed in the TCGA-LUAD to validate the prognosis (Fig. 5C). Thereafter, the calibration curves demonstrated a significant consistency between the predicted OS and the observed OS (Fig. 5D). Thereafter, the decision curve resulted from the 3- and 5-year analyses further indicated that the models incorporating clinical factors could predict the survival of LUAD patients better than independent predictors (Figs. 5E-F).

### GO and KEGG analyses of the two risk subgroups

**Fig. 6:**
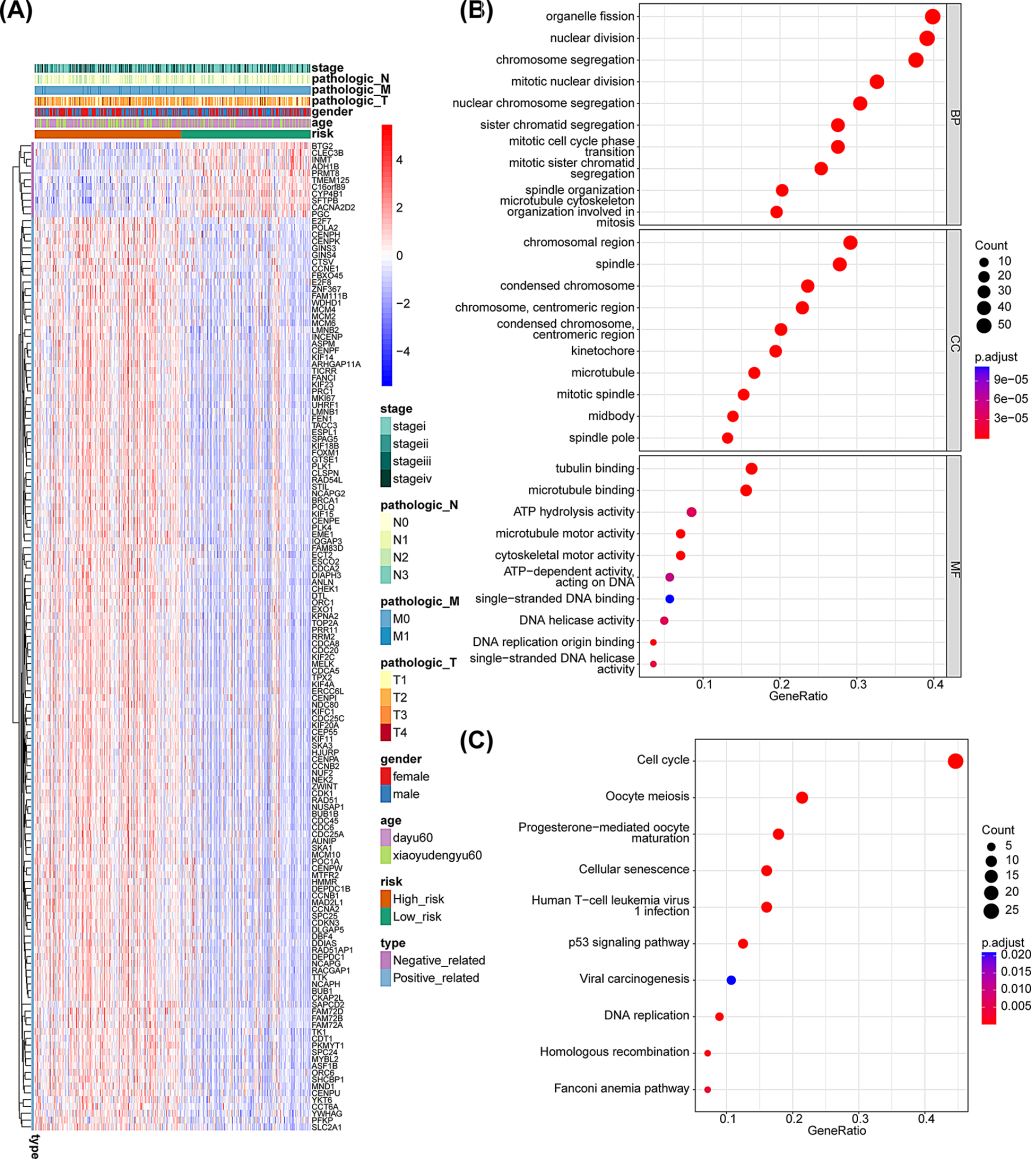
Function and pathway enrichment analysis. (**A**) Heatmap depicted DEGs between high- and low-risk subgroups. (**B**) Bubble plot of GO analysis on DEGs. (**C**) Bubble plot of KEGG analysis on DEGs

To elucidate the underlying molecular mechanisms of the prognostic risk model, we initially identified DEGs between the two risk subgroups in the TCGA-LUAD dataset. A total of 145 DEGs were identified (Fig. 6A). Then, GO and KEGG analysis were conducted to determine the effects of the 145 DEGs on the biological process. The GO results indicated that these DEGs were predominantly involved in terms such as organelle fission and chromosome segregation in BP; chromosomal region, spindle, and condensed chromosome in CC; tubulin binding and ATP hydrolysis activity in MF (Fig. 6B). In addition, in the KEGG pathways, a total of 10 signaling pathways, such as cell cycle and cellular senescence, were demonstrated to be significantly correlated with these DEGs (Fig. 6C).

### Analysis of tumor microenvironment

**Fig. 7:**
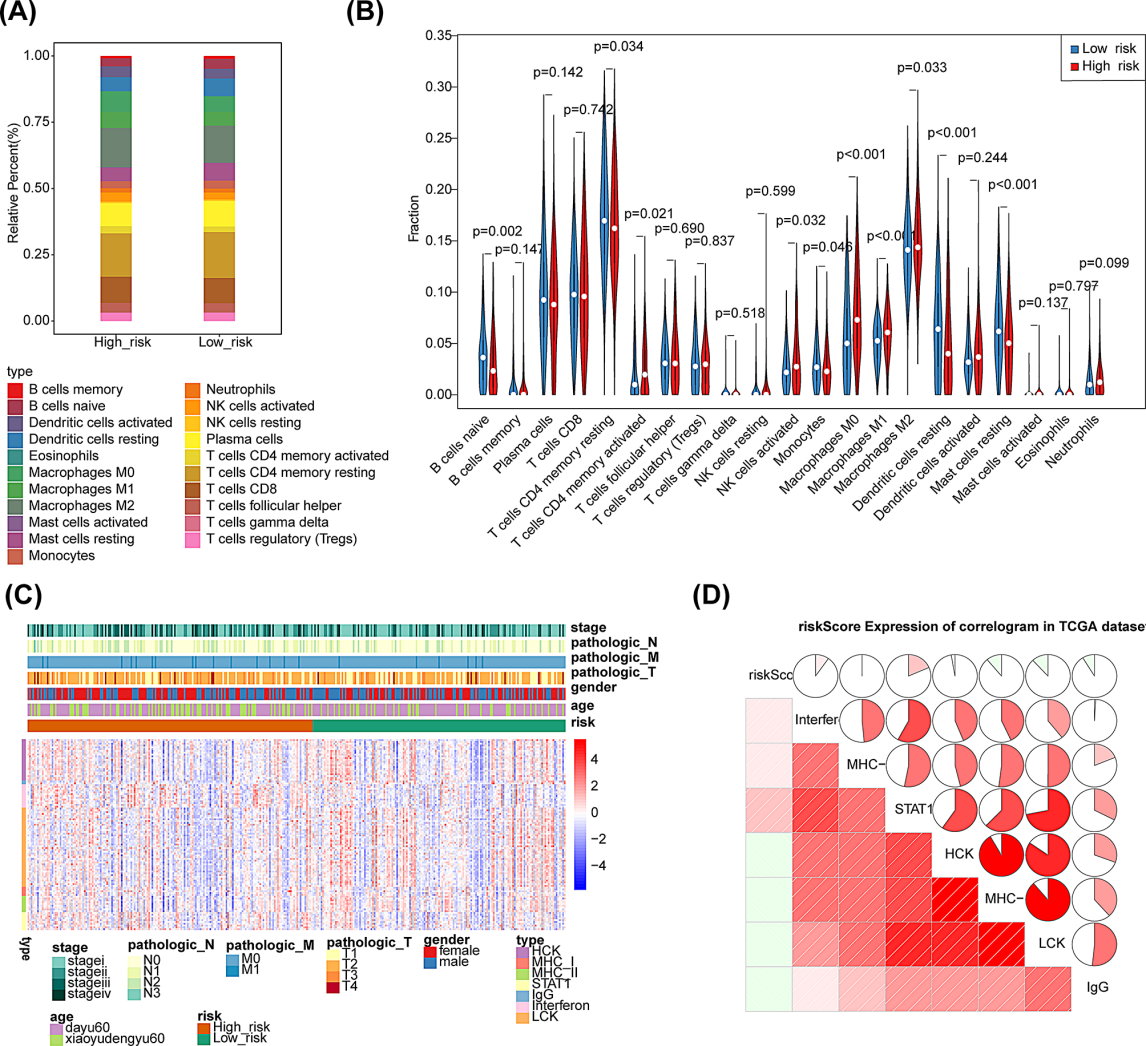
Tumor immune microenvironment analysis. (**A**) Percentage difference in immune cells between high- and low-risk subgroups. (**B**) Violin diagram for difference analysis of immune cells between the two risk subgroups. (**C**) Heatmap depicted differential expression of seven metagenic clusters among different subtypes. (**D**) Correlation analysis between the risk score and seven metagenic clusters

The infiltration of immune cells in the two risk subgroups of LUAD samples was estimated by the CIBERSORT method. The findings demonstrated that the percentage of immune infiltration in B cells naïve, T cells CD4 memory resting, T cells CD4 memory activated, NK cells activated, Monocytes, Macrophages M0, Macrophages M1, Macrophages M2, Dendritic cells resting, and Mast cells resting differed significantly between the two risk subgroups (Figs. 7A-B). Subsequently, the correlation between the risk score and seven metagenic clusters was examined, indicating that Interferon, MHCI, and STAT1 had positive correlations with the risk score, while LCK, HCK, IgG, and MHC-II had negative associations with the risk score (Figs. 7C-D).

### Correlation analysis of the risk model with immunotherapy and drug sensitivity

**Fig. 8:**
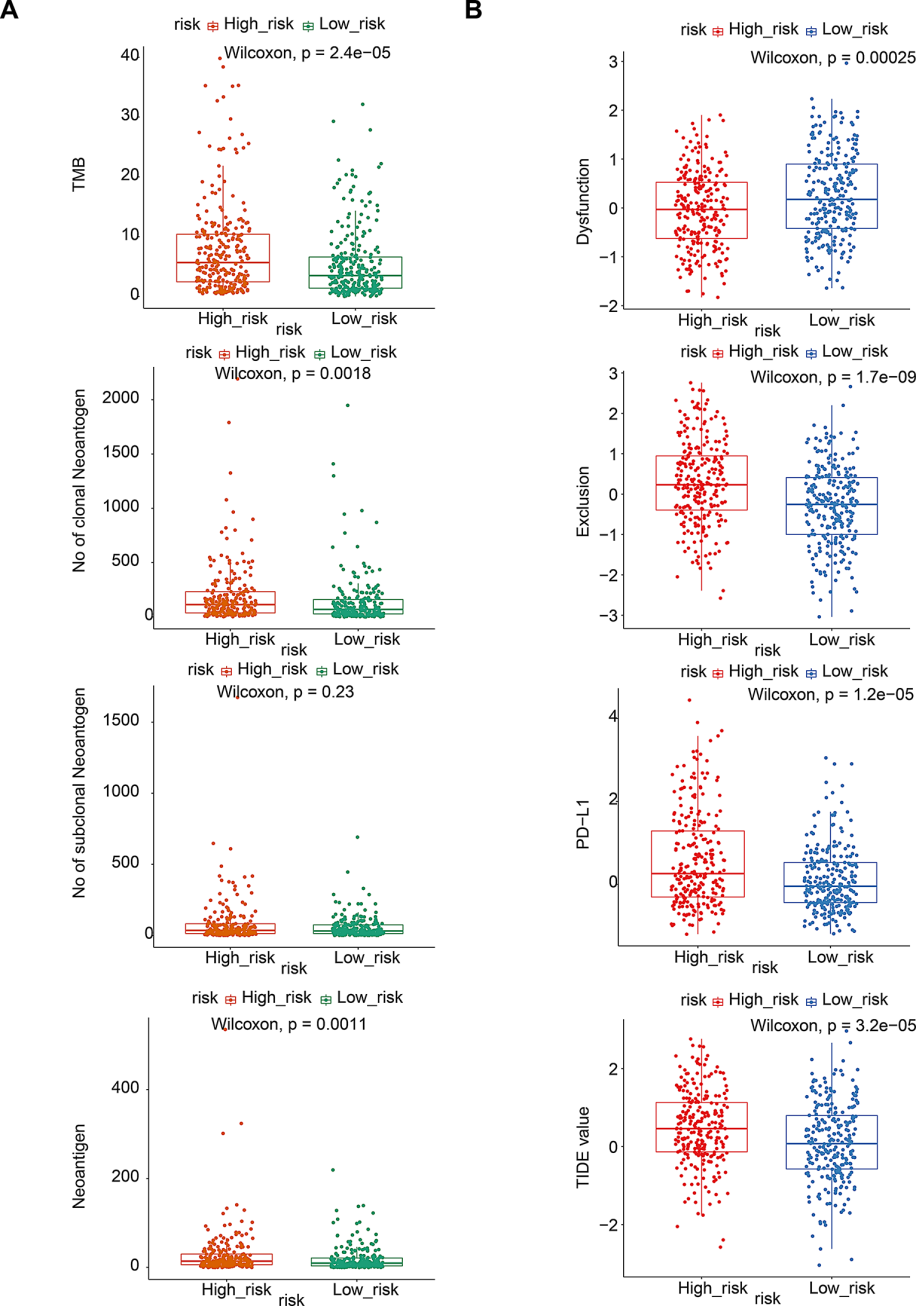
Immunotherapy analysis. (**A**) Difference analysis in TMB, neoantigen number, clonal neoantigen number and subclonal neoantigen number between the two risk subgroups. (**B**) Difference analysis in TIDE, Immune Dysfunction, Immune Exclusion and PD-L1 between the two risk subgroups

To investigate the association between the risk model and the immunotherapy response, we calculated the differences in tumor mutation burden (TMB), the number of neoantigen, the number of clonal neoantigen, and the number of subclonal neoantigen between the two risk subgroups. The results demonstrated that TMB was significantly higher in the low-risk group compared with the high-risk group (*P* < 0.05) (Fig. 8A). Additionally, we found that the TIDE, Immune Dysfunction, Immune Exclusion, and PD-L1 were significantly differentially expressed between the two risk subgroups (*P* < 0.05) (Fig. 8B). Subsequently, based on the GDSC database, the pRRophetic algorithm was applied to predict the sensitivity of LUAD to drugs in the two risk subgroups. IC50 results indicated that there were 68 drugs for which the drug sensitivity differed significantly between the two risk subgroups (Supplementary Fig. 2, Supplementary Table 2).

### Validation of the expression of the nine-risk model genes

**Fig. 9:**
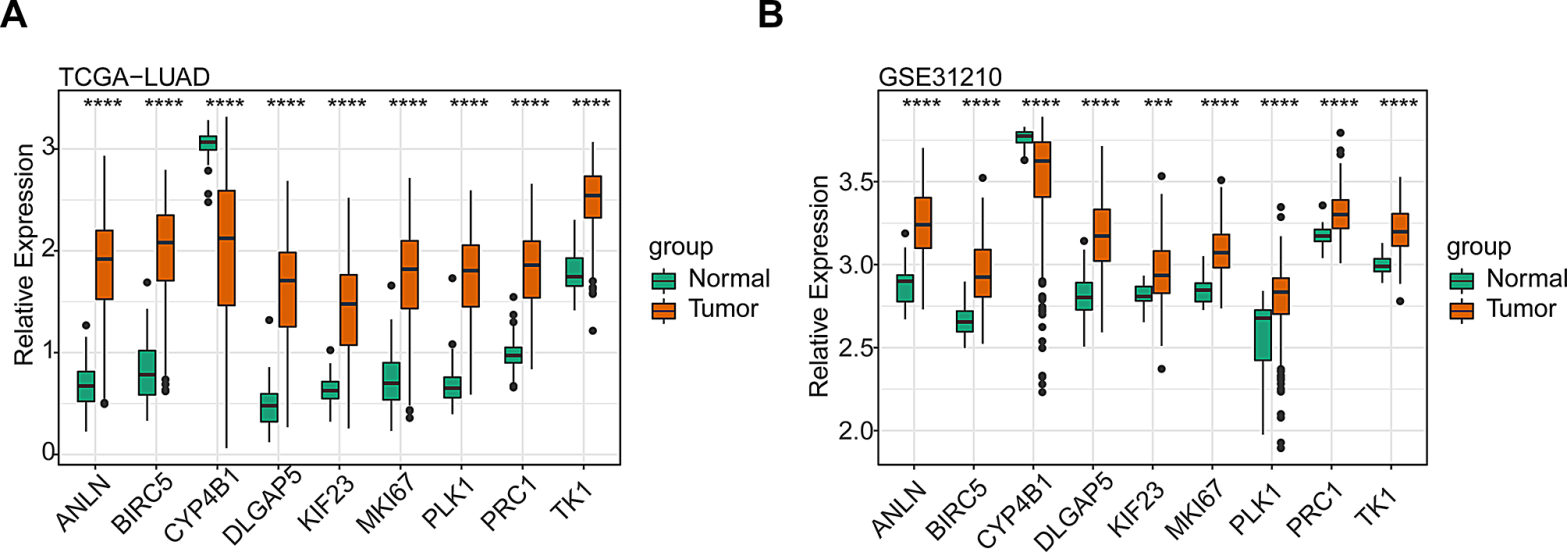
Validation on expressions of the nine prognostic genes. (**A**-**B**) Differences in gene expressions between LUAD and normal groups in TCGA-LUAD dataset (**A**) and GSE31210 dataset (**B**)

The expression of the nine risk model genes (*ANLN*,* PLK1*,* DLGAP5*,* PRC1*,* CYP4B1*,* MKI67*,* KIF23*,* BIRC5*, and *TK1*) were validated by using the Wilcoxon test in the TCGA-LUAD and GSE31210 datasets, respectively. The mRNA expression levels of *ANLN*,* PLK1*,* DLGAP5*,* PRC1*,* MKI67*,* KIF23*,* BIRC5*, and *TK1* were significantly elevated in LUAD tissues. In contrast, *CYP4B1* was significantly decreased compared with non-tumor tissues (Figs. 9A-B).

## Discussion

LUAD is the most frequent type of non-small cell lung cancer with high morbidity and mortality [1, 2]. Based on the close relationship between folic acid and lung cancer studied to date [17, 18], we hypothesized that folic acid metabolism and its associated genes might play an instrumental role in the progression of LUAD. The present study employed bioinformatics analysis to screen for folic acid-related biomarkers and established a FAMGs-based prognostic model for LUAD. A total of 77 FAMGs associated with LUAD were obtained by integrating and analyzing the transcriptome data from GEO and TCGA datasets. Ultimately, a prognostic model based on nine genes was successfully established by use of univariate and multivariate Cox regression analyses. Based on this model, patients were classified into high-risk and low-risk groups, with a significantly lower survival rate in the high-risk group. The model was confirmed to be stable and possess better predictive performance.

GO functional and KEGG pathway enrichment analyses were conducted to elucidate the underlying molecular mechanisms of the prognostic risk model. The results indicated that 145 DEGs were involved in the cellular component of condensed chromosome, the biological process of cell division, and also several signaling pathways, such as cell cycle, cellular senescence, etc. Studies have demonstrated that folic acid deficiency leads to inappropriate DNA methylation, disturbs the synthesis of 2’-deoxythymidine- 5’monophophate (dTMP), and increases the misincorporation of uracil [11, 36]. These events might further result in alterations in DNA methylation, disruption of DNA integrity, disruption of DNA repair, and even chromosome aberration [21, 37]. These aberrations in DNA are considered to boost carcinogenesis through modifying the expression of critical tumor suppressor genes and proto-oncogenes, and affecting some major events such as cell division, cell cycle and chromosome formation, and ultimately cause the development of LUAD [38]. This may be an underlying mechanism for the progression of LUAD induced by folic acid deficiency and related dysmetabolisms.

Based on the expression patterns of FAMGs, we obtained 9 DEGs associated with prognosis (*ANLN*,* PLK1*,* DLGAP5*,* PRC1*,* CYP4B1*,* MKI67*,* KIF23*,* BIRC5*,* and TK1*). *ANLN* is an actin-binding protein and has been identified as a critical factor in cell proliferation and migration, particularly in cytokinesis [39]. Studies have demonstrated that up-regulation of ANLN was correlated with several cancers, including those of the liver, pancreas, breast, bladder, esophageal, and lung [39–41]. Singh et al. discovered that *ANLN* was significantly up-regulated in LUAD and had a mutation frequency of 3.73% [42]. It was associated with advanced stages, clinicopathological parameters, the deterioration of relapse-free survival (RFS), and OS, thereby identifying its oncogenic and prognostic potential [42]. *PLK1* is an evolutionary conserved Ser/Thr kinase and is widely recognized for its effects on cell cycle regulation as well as in DNA damage response, autophagy, apoptosis, and cytokine signaling. Studies have found that *PLK1* is overexpressed in several cancers including LUAD, and is associated with a poor prognosis [43, 44]. Kong et al. demonstrated that *PLK1* accelerated the progression of LUAD by amplifying MAPK pathway [45]. *DLGAP5* belongs to the disc large-associated protein (DLGAP) family. It plays important roles in tumorgenesis, metastasis, and drug resistance as well. Studies found that knockdown of *DLGAP5* significantly inhibited the proliferation and invasion of various tumors. *DLGAP5* also predicts poor prognosis and immunotherapy response in LUAD [46]. *PRC1* is correlated with Polycomb group (PcG) genes. The dysregulation of *PRC1* has been associated with the initiation and progression of human cancers [47]. Zhu et al. have reported a notable increase in *PRC1* expression in LUAD, which may be correlated with cell cycle, cytokinesis, and the p53 signaling pathway [48]. *CYP4B1* is a specific member of the P450 family, and is predominantly expressed in lung tissues. *CYP4B1* and its single nucleotide polymorphisms have been found to be involved in several cancers [49]. Liu et al. reported that the mRNA level of *CYP4B1* was significantly decreased in LUAD samples, and its low expression was an independent prognostic indicator for a decreased overall survival (OS) and recurrence-free survival (RFS) [50]. Both *MKI67* and *KIF23* are proliferation-related genes. They are both well-known for their significant clinical and prognostic value in a wide range of tumors, including breast cancer, hepatocellular carcinoma, gastric cancer, glioma, and lung cancer [51–54]. *BIRC5*, also known as Survivin, is a popular target for cancer treatment, and it is found to be the fourth most upregulated mRNA in the human cancer transcriptome [55]. *TK1* is a cytosolic enzyme involved in pyrimidine metabolism for DNA synthesis and DNA damage. It is also a proliferation marker and has been investigated as a diagnostic tool and prognostic factor for evaluating cancer treatment and disease progression [56]. In the present study, except for the reduced expression of *CYP4B1*, the other eight DEGs were significantly increased in LUAD tumor tissues compared with normal tissues. The disturbance of folic acid-related metabolisms may affect expression patterns of these genes and finally leads to the occurrence of LUAD. Nevertheless, further research is requisite for detailed mechanisms.

Tumours consist of a complex microenvironment composed of immune, stromal, and cancer cells. The tumor microenvironment is often characterized by hypoxic, highly oxidative, acidic, and nutrient-poor conditions due to the requirement for rapid proliferation [57]. In this study, we investigated the effects of immune microenvironment status on the prognosis of LUAD. It was found that the tumor immune microenviroment has substantial influences on tumor progression and metastases. Many immune cells, including lymphocytes (TLCs), natural killer (NK) cells, dendritic cells (DCs), neutrophils, and macrophages were involved and associated with patients’ survival and relapse.

We also investigated the response of immunotherapy between the two risk subgroups. The results indicated that TMB was significantly decreased in the low-risk subgroup, demonstrating a better overall survival. Besides, expressions of checkpoint PD-L1 were significantly increased in the high-risk subgroup. Wang et al. also found an up-regulation of TMB and PD-L1 expression in high-risk LUAD patients, indicating that these patients may benefit more from immune checkpoint blockade therapy [58]. Furthermore, we identified substantial differences in drug sensitivity for up to 164 drugs between the two risk groups. Notably, low-risk patients exhibited superior drug sensitivity, suggesting a more favorable prognosis. Consequently, further clinical investigations are necessary to validate these findings. We may also analyze gender and pathological M as potential concomitant variables in future work, which might assist in determining other potential interacting factors and better understanding their roles in immunotherapy or disease prognosis.

The present study identified the potential role of FAMGs in LUAD through bioinformatics analysis. However, the results are merely theoretical and lack clinical as well as in-depth functional validations. Therefore, more clinical or multicenter studies are needed to validate the results, and additional experiments are necessary to investigate the mechanisms of the nine prognostic genes and related signaling pathways in the progression of LUAD. In our forthcoming study, mRNA and protein expression levels for the prognostic nine genes will be collected and verified through animal and cell experiments. Additionally, the molecular mechanisms by which these genes interact with other signaling pathways and biomarkers, as well as their impact on the development and progression of LUAD will also be investigated. This will provide new clues and strategies for the precise diagnosis and individualized treatment of LUAD.

## Conclusion

In conclusion, the current study is one of the first ones to focus on the role of folic acid metabolism-related genes (FAMGs) in LUAD by integrating and analyzing LUAD transcriptome data from GEO and TCGA datasets. We concluded that FAMGs can function as promising biomarkers to predict the prognosis and treatment strategy of LUAD.

## Electronic supplementary material

Below is the link to the electronic supplementary material.

Supplementary Material 1: Supplementary Fig. 1. Kaplan-Meier (K-M) survival analysis of the 25 DEGs.

Supplementary Material 2: Supplementary Fig. 2. Drug sensitivity analysis of the 68 drugs.

Supplementary Material 3: Supplementary Table 1. Univariate Cox regression analysis and K-M analysis of 25 DEGs.

Supplementary Material 4: Supplementary Table 2. IC50 values for all drugs (Attached as a separate document in excel form).

## Data Availability

The data availability statement is provided within the manuscript.

## Abbreviations

Abbreviations: Full name in English
LUAD: Lung Adenocarcinoma
FAMGs: Folic Acid Metabolism-related Genes
GEO: Gene Expression Omnibus
TCGA: The Cancer Genome Atlas
DEGs: Differentially Expressed Genes
OS: Overall Survival
ROC: Receiver Operating Characteristic
SAdoMet: S-Adenosylmethionine
FRA: Folic acid Receptor Alpha
GPI: Glycosyphosphatidylinositol
KEGG: Kyoto Encyclopedia of Genes and Genomes
AMIGO2: Adhesion Molecule with Ig-like domain 2
GO: Gene Ontology
ROC: Receiver Operating Characteristic
K-M: Kaplan-Meier
TMB: Tumour Mutation Burden
GDSC: Drug Sensitivity in Cancer
CC: Cellular Component
BP: Biological Process
MF: Molecular Function
NK: Natural Killer
DCs: Dendritic Cells
TMB: Tumour Mutation load
