# Supplementary figures and images for: Characteristics of folic acid metabolism-related genes unveil prognosis and treatment strategy in lung adenocarcinoma
Source: BMC Pulm Med. 2025 May 22;25:255. doi: 10.1186/s12890-025-03694-x (PMC12101037; doi:10.1186/s12890-025-03694-x)

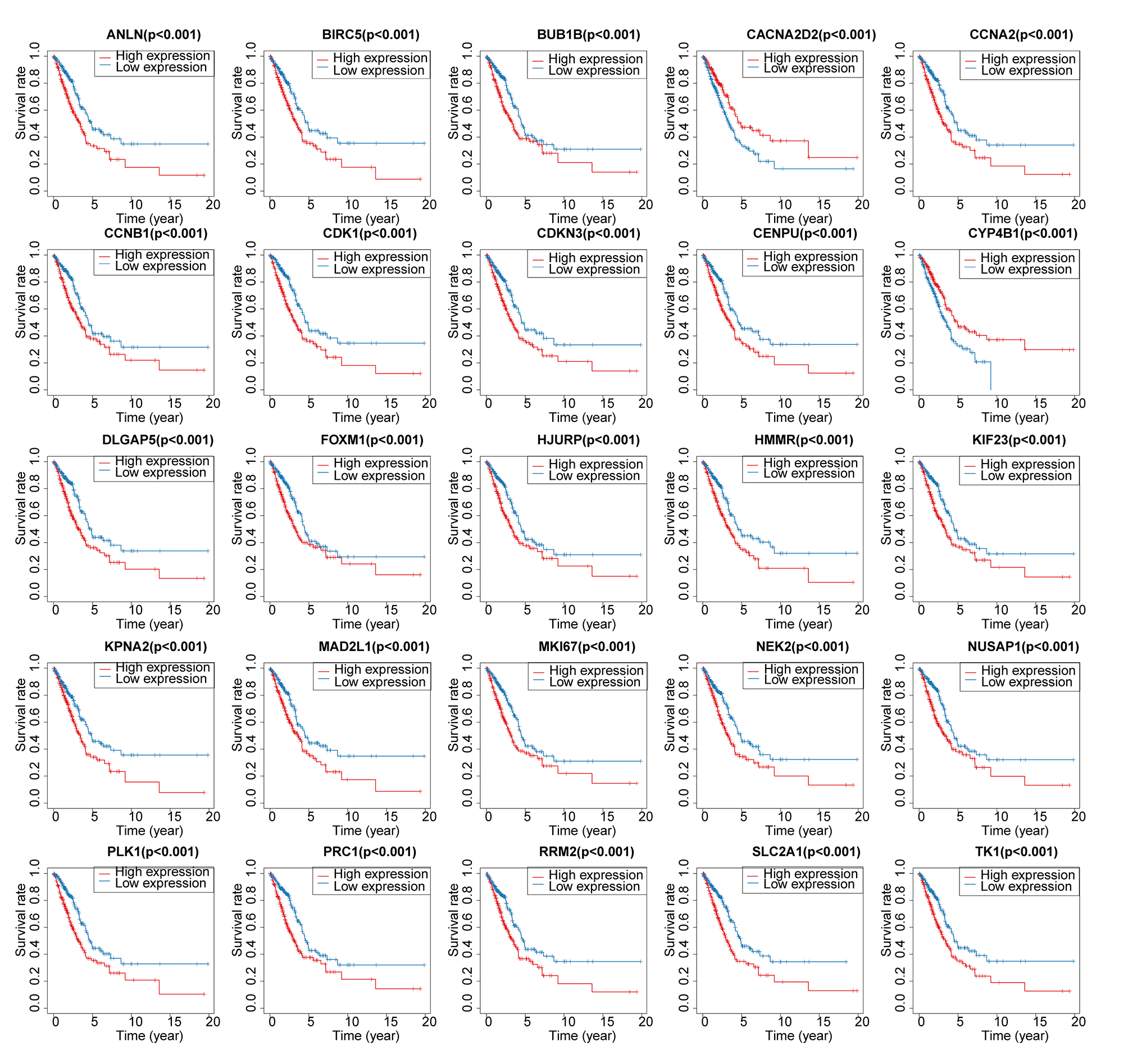

Supplement: Supplementary file 1 — Supplementary Material 1: Supplementary Fig. 1. Kaplan-Meier (K-M) survival analysis of the 25 DEGs. [file 12890_2025_3694_MOESM1_ESM.tif]

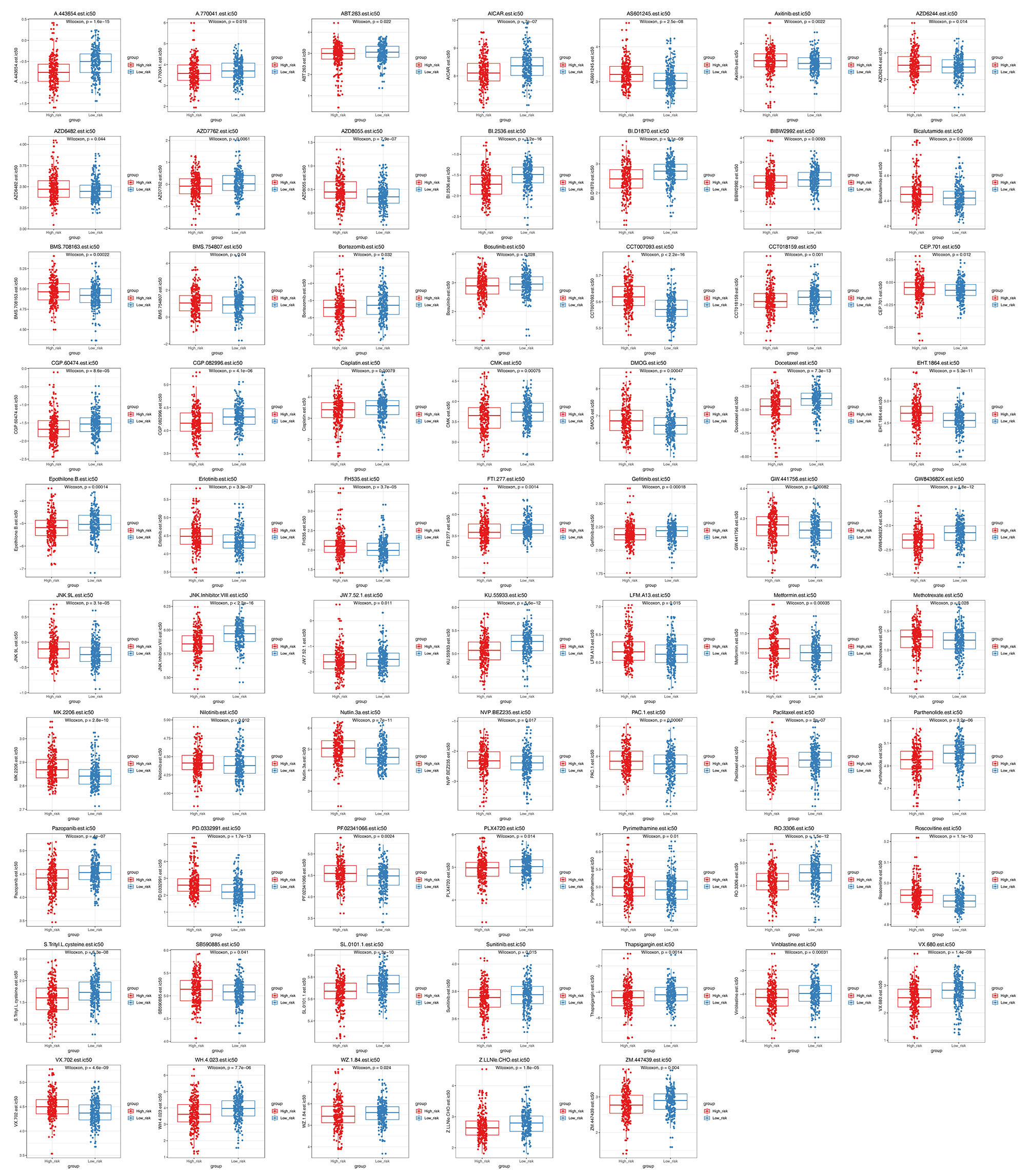

Supplement: Supplementary file 2 — Supplementary Material 2: Supplementary Fig. 2. Drug sensitivity analysis of the 68 drugs. [file 12890_2025_3694_MOESM2_ESM.tif]
