# Supplementary material for: Characteristics of folic acid metabolism-related genes unveil prognosis and treatment strategy in lung adenocarcinoma
Source: BMC Pulm Med. 2025 May 22;25:255. doi: 10.1186/s12890-025-03694-x (PMC12101037; doi:10.1186/s12890-025-03694-x)
Supplement: Supplementary file 4 — Supplementary Material 4: Supplementary Table 2. IC50 values for all drugs (Attached as a separate document in excel form). [file 12890_2025_3694_MOESM4_ESM.docx]

**Supplementary Table 1.** Univariate Cox regression analysis and K-M analysis of 25 DEGs

| id | HR | HR.95L | HR.95H | pvalue | KM |
| --- | --- | --- | --- | --- | --- |
| ANLN | 1.385192098 | 1.224892798 | 1.566469451 | 2.07E-07 | 4.79E-05 |
| BIRC5 | 1.22883885 | 1.090920604 | 1.384193234 | 0.000692162 | 0.000446828 |
| BUB1B | 1.305363646 | 1.123476941 | 1.516697128 | 0.000500059 | 0.000533259 |
| CACNA2D2 | 0.84576913 | 0.772940275 | 0.925460147 | 0.000266256 | 0.000980669 |
| CCNA2 | 1.322741958 | 1.159514782 | 1.508946945 | 3.15E-05 | 6.56E-05 |
| CCNB1 | 1.337691031 | 1.167649433 | 1.532495323 | 2.74E-05 | 0.00065663 |
| CDK1 | 1.277528862 | 1.120186903 | 1.456971145 | 0.000259748 | 2.97E-05 |
| CDKN3 | 1.320716081 | 1.153600099 | 1.512041275 | 5.58E-05 | 0.000428722 |
| CENPU | 1.320107454 | 1.131288974 | 1.540440799 | 0.000421368 | 0.000460317 |
| CYP4B1 | 0.858879456 | 0.800138664 | 0.921932601 | 2.57E-05 | 0.000751076 |
| DLGAP5 | 1.355513825 | 1.182258732 | 1.55415873 | 1.30E-05 | 9.80E-05 |
| FOXM1 | 1.287935904 | 1.139438103 | 1.455786751 | 5.16E-05 | 0.000981029 |
| HJURP | 1.332555457 | 1.162505364 | 1.527480303 | 3.76E-05 | 0.000612826 |
| HMMR | 1.40804654 | 1.208113916 | 1.641066321 | 1.19E-05 | 0.000193983 |
| KIF23 | 1.335521393 | 1.141308023 | 1.562783538 | 0.000308002 | 0.000347319 |
| KPNA2 | 1.358011649 | 1.163869045 | 1.584538782 | 0.00010112 | 0.000984724 |
| MAD2L1 | 1.309469636 | 1.119503959 | 1.531670089 | 0.000747381 | 0.000452034 |
| MKI67 | 1.29945551 | 1.136014328 | 1.486411376 | 0.000133769 | 0.000239021 |
| NEK2 | 1.304351174 | 1.140432712 | 1.491830221 | 0.000105428 | 0.000414606 |
| NUSAP1 | 1.277640534 | 1.107239981 | 1.474265166 | 0.000794243 | 0.000230086 |
| PLK1 | 1.409124951 | 1.219063699 | 1.62881819 | 3.49E-06 | 7.38E-05 |
| PRC1 | 1.387366917 | 1.192141085 | 1.614563064 | 2.32E-05 | 5.72E-05 |
| RRM2 | 1.326276106 | 1.167697855 | 1.506389946 | 1.39E-05 | 0.000496459 |
| SLC2A1 | 1.263190455 | 1.136775402 | 1.403663487 | 1.41E-05 | 3.58E-05 |
| TK1 | 1.313518262 | 1.149043233 | 1.501536388 | 6.46E-05 | 0.000242689 |
